# SOCS5 inhibition induces autophagy to impair metastasis in hepatocellular carcinoma cells via the PI3K/Akt/mTOR pathway

**DOI:** 10.1038/s41419-019-1856-y

**Published:** 2019-08-13

**Authors:** Mao Zhang, Shihai Liu, Mei-Sze Chua, Haoran Li, Dingan Luo, Sheng Wang, Shun Zhang, Bing Han, Chuandong Sun

**Affiliations:** 1 Department of Hepatobiliary and Pancreatic Surgery, Affiliated Hospital of Qingdao University, Qingdao, Shandong P. R. China; 2 Medical Animal Laboratory, Affiliated Hospital of Qingdao University, Qingdao, Shandong P. R. China; 3 Asian Liver Center, Department of Surgery, Stanford University School of Medicine, Stanford, CA USA; 4 Department of Colorectal Surgery, Fudan University Shanghai Cancer Center, Shanghai, P. R. China

**Keywords:** Metastasis, Metastasis

## Abstract

SOCS5 is a member of the suppressor of cytokine signaling (SOCS) protein family with important yet incompletely understood biological functions in cancer. In hepatocellular carcinoma (HCC), controversial tumor-promoting and tumor-suppressive roles of SOCS5 have been reported. Our study aims to unravel novel functions of SOCS5 in HCC, especially that affecting metastasis. We examined the expression levels of SOCS5 in HCC using publicly available datasets, and in our patient cohort, using quantitative real-time PCR, western blotting, and immunohistochemistry. The association of SOCS5 expression with clinical pathological data of HCC patients was examined and that with the mTOR pathway was predicted. We further studied the effects of SOCS5 on PI3K/Akt/mTOR activity; HCC cell autophagy, migration, and invasion; and HCC cell metastasis in vitro and in vivo. We observed that SOCS5 was significantly overexpressed in HCC tissues, compared to adjacent non-tumor liver tissues, in both the public datasets and in our patient cohort. SOCS5 overexpression was significantly and inversely correlated with HCC patient prognosis. Moreover, SOCS5 overexpression promoted HCC cell migration and invasion in vitro by inactivating PI3K/Akt/mTOR-mediated autophagy. Conversely, SOCS5 inhibition suppressed HCC cell migration and invasion in vitro by activating PI3K/Akt/mTOR-mediated autophagy. Dual inhibition of SOCS5 and mTOR further enhanced autophagy and the subsequent anti-metastatic effects on HCC cells. In vivo, stable knockdown of SOCS5 reduced HCC cell metastasis. Overall, our study revealed a novel metastasis-promoting function of SOCS5 in HCC, acting via the PI3K/Akt/mTOR-mediated autophagy pathway. Combined inhibition of SOCS5 and mTOR may be a potential therapeutic approach to inhibit HCC metastasis and prolong patient survival.

## Introduction

Hepatocellular carcinoma (HCC) is the fourth leading cause of cancer mortality worldwide and the third leading cause of cancer mortality in China^1,2^. Although significant developments have been made recently in the diagnosis and treatment of HCC, the prognosis of HCC remains poor due to the high rates of recurrence and metastasis^3^. Unfortunately, the mechanisms underlying HCC metastasis remain poorly understood. Therefore, an improved understanding of the molecular mechanisms underlying HCC metastasis have the potential to help develop new therapeutic strategies for HCC, which are urgently needed to prolong patient survival.

Members of the suppressor of cytokine signaling (SOCS) protein family play key roles in the regulation of cytokine signaling^4–6^ and aberrant expression levels of SOCS members have been reported to contribute to disease development and progression^7^. Whereas certain members of SOCS, such as SOCS1–3 and cytokine-inducible SH2-containing protein (CIS), have been extensively studied^8–11^, the biological effects of SOCS5 remain largely unknown. Yoon et al. reported that SOCS5 was upregulated in the three liver cancer tissues^7^. The data from this limited sample set require validation in a larger liver cancer patient cohort. In a recent study using liver cancer cell lines, SOCS5 was shown to have tumor-suppressive role^12^. Our study aims to fill in the gaps in understanding the contribution of SOCS5 to HCC development and/or progression, which may form the basis for developing new therapeutic strategies for HCC.

Tumor metastasis causes >90% of cancer deaths, and there is increasing evidence that autophagy is intricately involved in tumor metastasis^13,14^. Autophagy is a highly conserved catabolic process, which maintains cellular homeostasis by targeting damaged proteins or organelles to lysosomal compartments for degradation^15,16^; this process is important in both normal human physiology and diseases, including cancer^17^. In HCC, α-fetoprotein (AFP)-mediated inhibition of autophagy was associated with enhanced cell proliferation, migration, and invasion, likely involving activation of the phosphoinositide-3 kinase (PI3K)/Akt/mammalian target of rapamycin (mTOR) signaling pathway^14^. While autophagy and the PI3K/Akt/mTOR pathway appear to play critical roles in HCC metastasis, it is unknown whether SOCS5 may be a mediator in these processes.

In this study, we systematically studied the expression of SOCS5 in two large independent cohorts of HCC patients and determined the association of SOCS5 expression with clinical pathological characteristics, including patient survival. We then examined the functional role(s) of SOCS5 in HCC cell migration, invasion, autophagy, and metastasis using HCC cell line and animal models. Our data highlight the novel role of SOCS5 in mediating autophagy via the PI3K/Akt/mTOR pathway, with resulting effects on HCC cell metastasis in vitro and in vivo.

## Materials and methods

### Patient samples and immunohistochemistry (IHC)

This study was performed with approval from the Ethics Committee at the Affiliated Hospital of Qingdao University. Paraffin-embedded, histopathologically, and clinically diagnosed HCC samples (*n* = 102) were collected at the Affiliated Hospital of Qingdao University from January 2013 to July 2014. The clinicopathological characteristics of the samples are shown in Table 1. An additional 43 frozen HCC tissues and matched adjacent non-tumor liver tissues were collected from patients who underwent surgery at the Affiliated Hospital of Qingdao University in 2017.

**Table 1:** Correlation of SOCS5 expression with clinicopathological features in HCC

Variables	Number	SOCS5, *n* (%)		*χ* ^2^	*P* value
		Low	High		
Gender				0.874	0.350
Male	84	52 (61.9)	32 (38.1)		
Female	18	9 (50.0)	9 (50.0)		
Age (years)				2.268	0.132
<50	20	9 (45.0)	11 (55.0)		
≥50	82	52 (63.4)	30 (36.6)		
Alcoholism				0.174	0.677
Yes	30	17 (56.7)	13 (43.3)		
No	72	44 (61.1)	28 (38.9)		
Liver cirrhosis				3.293	0.070
Yes	79	51 (64.6)	28 (35.4)		
No	23	10 (43.5)	13 (56.5)		
AFP level (ng/L)				3.972	**0.046**
≤400	71	47 (66.2)	24 (33.8)		
>400	31	14 (45.2)	17 (54.8)		
ALT level (U/L)				0.014	0.906
≤60	79	47 (59.5)	32 (40.5)		
>60	23	14 (60.9)	9 (39.1)		
AST level (U/L)				0.152	0.696
≤42	75	44 (58.7)	31 (41.3)		
>42	27	17 (63.0)	10 (37.0)		
Tumor number				1.378	0.240
Single	85	53 (62.4)	32 (37.6)		
Multiple	17	8 (47.1)	9 (52.9)		
Tumor size (cm)				7.135	**0.008**
≤5	70	48 (68.6)	22 (31.4)		
>5	32	13 (40.6)	19 (59.4)		
Microvascular invasion				4.178	**0.041**
Yes	33	15 (45.5)	18 (54.5)		
No	69	46 (66.7)	23 (33.3)		
Portal vein invasion				4.374	**0.036**
Yes	8	2 (25.0)	6 (75.0)		
No	94	59 (62.8)	35 (37.2)		
TNM stage				12.270	**0.002**
I	61	44 (72.1)	17 (27.9)		
II	28	14 (50.0)	14 (50.0)		
III	13	3 (23.1)	10 (76.9)		

Statistical data on SOCS5 expression in relation to clinicohistopathologic features for surgical HCC specimens. *P* values were calculated using chi-square test. Bold numbers indicate significant differences (*P* < 0.05)

*SOCS5* suppressor of cytokine signaling 5, HCC hepatocellular carcinoma, *AFP* α-fetoprotein, *ALT* alanine aminotransferase, *AST* aspartate aminotransferase, *TNM* tumor, node, metastasis

The SOCS5 protein expression levels in 102 paraffin-embedded HCC tissues were examined by IHC as described previously^18^. Scoring of IHC staining was based on the intensity of staining (0 = negative, 1 = weak, 2 = medium, or 3 = strong) and the percentage of positively stained cells within the observed field (0 = 0%, 1 = 1–25%, 2 = 26–50%, and 3 = ≥51%). Final staining scores were determined by multiplying the intensity and percentage scores and ranged from 0 to 9. Low expression was defined as having final scores <4, and high expression was defined as final scores of 4–9. Expression level of SOCS5 was correlated with clinical pathological characteristics of HCC patients using chi-square test.

### Bioinformatics analysis

To investigate the clinical significance of SOCS5 in HCC, we retrieved and analyzed the mRNA expression of SOCS5 in HCC tissues and non-tumor liver tissues using published data from Oncomine (https://www.oncomine.org/resource/main.html). Subsequently, we analyzed the relationship of SOCS5 expression with HCC prognosis by using Oncolnc (http://www.oncolnc.org). We also acquired gene expression data from TCGA (https://portal.gdc.cancer.gov/projects/TCGA-LIHC) and analyzed the data using Gene Set Enrichment Analysis (GSEA).

### Cell culture and reagents

Normal liver cell line (L-02) and liver cancer cell lines (HepG2, PLC/PRF/5, Hep3B, and Huh7) were purchased from a cell bank at the Chinese Academy of Sciences (Shanghai, China). L-02 cells were cultured in Roswell Park Memorial Institute (RPMI)-1640 with 20% fetal bovine serum (FBS) and 1% P/S (100 IU/mL penicillin and 100 IU/mL streptomycin); HepG2, PLC/PRF/5, and Hep3B were cultured in Minimum Essential Medium with 10% FBS and 1% P/S; Huh7 cells were cultured in Dulbecco’s modified Eagle’s medium supplemented with 10% FBS and 1% P/S; and all cells were deposed in a humidified atmosphere with 5% CO_2_ at 37 °C.

Dimethyl sulfoxide, 3-methyladenine (3-MA) and Bafilomycin A1 (Baf A1) were purchased from Sigma-Aldrich (St. Louis, MO). Rapamycin (Rap) was purchased from MCE (Monmouth Junction, NJ).

### Transfection

Cells were transfected with plasmids expressing SOCS5 (GV141-SOCS5, Genechem, Shanghai, China), plasmids expressing empty vector (GV141-Vector), small interfering RNAs (siRNAs) against SOCS5 (siSOCS5; GenePharma, Shanghai, China; Supplementary Table 1), negative control (siNC), or with GFP-RFP-LC3 using Lipofectamine 2000 (Invitrogen, Carlsbad, CA) according to the manufacturer’s instructions. Cells were transfected with SOCS5 siRNAs for 12, 24, or 48 h and harvested for subsequent experiments.

### RNA extraction and quantitative real-time PCR (qPCR)

Total RNA from cultured cells and frozen tissues was extracted with TRIzol (Invitrogen, Carlsbad, CA). cDNA synthesis was performed using the PrimeScript™ RT Kit (TaKaRa, Otsu, Japan). SYBR Premix EX Taq™ (TaKaRa, Otsu, Japan) was used for qPCR on an FTC-3000p Real-Time PCR system (Funglyn Biotech, Shanghai, China). Relative gene expression was determined by the comparative 2^−ΔΔCT^ method. The PCR primers used are listed in Supplementary Table 1.

### Western blotting analysis

Western blotting analysis of protein expression was performed as described previously^18^. Briefly, protein lysates (20 µg) were separated using sodium dodecyl sulfate-polyacrylamide gel electrophoresis, and target proteins were detected by western blotting with antibodies against SOCS5 (1:500) and β-actin (1:50,000). Other antibodies used in this study are listed in Supplementary Table 1.

### Immunofluorescence

Cells were transfected with GFP-RFP-LC3 and GV141-Vector/GV141-SOCS5 or GFP-RFP-LC3 and siNC/siSOCS5 #3. Then the treated cells were inoculated onto glass slides and fixed with 4% paraformaldehyde (Boster, Wuhan, China) for 15 min. The fixed cells were treated with 5% bovine serum albumin (Sigma-Aldrich, St. Louis, MO) for 30 min and stained with 4′,6-diamidino-2-phenylindole (Beyotime, Shanghai, China) for 7 min, after which the coverslips were air-dried and mounted. Cells were viewed using a confocal laser-scanning microscope (Leica, Wetzlar, Germany). For quantification, we counted the numbers of GFP-RFP-LC3 dots in five independent visual fields.

### Electron microscopy

Cells were fixed with 2.5% glutaraldehyde (Solarbio, Beijing, China) for 4 h at 4 °C, washed with phosphate-buffered saline (PBS), and post-fixed with 1% OsO_4_ buffer for 2 h at 4 °C. The cells were then washed and dehydrated in a graded series of ethanol solutions and embedded in Epon812 epoxy resin. Ultrathin (90 nm) sections were collected on copper grids, double-stained with 1% uranyl acetate and 0.2% lead citrate, and examined by a JEOL-1200EX transmission electron microscope (Beijing, China).

### Cell migration and invasion assays

Cell migration and invasion were assessed using 8-μm-pore transwell compartments (Corning, NY). For migration assays, 5 × 10^4^ cells were suspended in serum-free medium in the upper compartment. After cells were incubated at 37 °C for 24 h, the translocated cells were stained with 0.5% crystal violet for 20 min at room temperature. For invasion assays, Matrigel (BD Biosciences, San Jose, CA) was added to each well according to the manufacturer’s instructions before 2 × 10^5^ cells were suspended in the upper compartment. After cells were incubated at 37 °C for 24 h, the translocated cells were incubated with 0.5% crystal violet for 20 min at room temperature. For quantification, cells were counted under a light microscope (Nikon, Tokyo, Japan) in five fields (upper, lower, middle, left, right; at ×40 magnification).

### Wound-healing assay

Cell migration was also assessed using the wound-healing assay. Briefly, a wound was generated in a 6-well plate by scratching the surface with a 200 μL pipette tip. The wounded areas were photographed under a light microscope (Nikon, Tokyo, Japan) at the time the wound was created (0 h) and at 24 h after. The percentage of wound healing was calculated using the following formula: [1 − (empty area 24 h/empty area 0 h)] × 100.

### Lentivirus construction and infection of cell lines

The lentiviral vectors encoding short hairpin RNAs (shRNAs) targeting SOCS5 and scrambled shRNA were purchased from Genomeditech (Shanghai, China; Supplementary Table 1) and designated as LV-shNC and LV-shSOCS5, respectively. Briefly, cells were infected with lentiviral particles in the presence of 5 μg/mL polybrene (Genomeditech, Shanghai, China); after 48 h, the supernatant was substituted with complete culture medium, and the transduced cells were selected for 7 days with 1 μg/mL puromycin (Solarbio, Beijing, China).

### Animal experiments

Animal experiments were conducted in strict accordance with the principles approved by the Committee on the Ethics of Animal Experiments of Qingdao University. Female BALB/c nude mice (4–6-week old) were purchased from Beijing Vital River Laboratory Animal Technology (Beijing, China). For metastasis observations, 12 BALB/c nude mice were randomly divided into two groups. Mice were injected with 2 × 10^6^ Huh7^LV-shNC^ cells or 2 × 10^6^ Huh7^LV-shSOCS5^ cells suspended in 200 μL PBS via the tail veins. After 6 weeks, all the nude mice were sacrificed, and the lungs were excised and embedded in paraffin for hematoxylin and eosin staining or IHC staining. Antibodies used for IHC used are listed in Supplementary Table 1.

### Statistical analysis

All statistical analyses were carried out using the Graphpad Prism 7.0 Software (GraphPad, La Jolla, CA). Categorical data were analyzed using chi-square (*χ*^2^) test. Cox proportional hazard model was used for univariate and multivariate survival analysis. Survival curves were plotted using the Kaplan–Meier method and compared using log-rank test. Analysis of variance and Student’s *t* test were used for comparison among groups. *P* < 0.05 was considered statistically significant.

## Results

### SOCS5 mRNA and protein expression levels are significantly overexpressed in HCC compared to non-tumor liver tissues

**Fig. 1:**
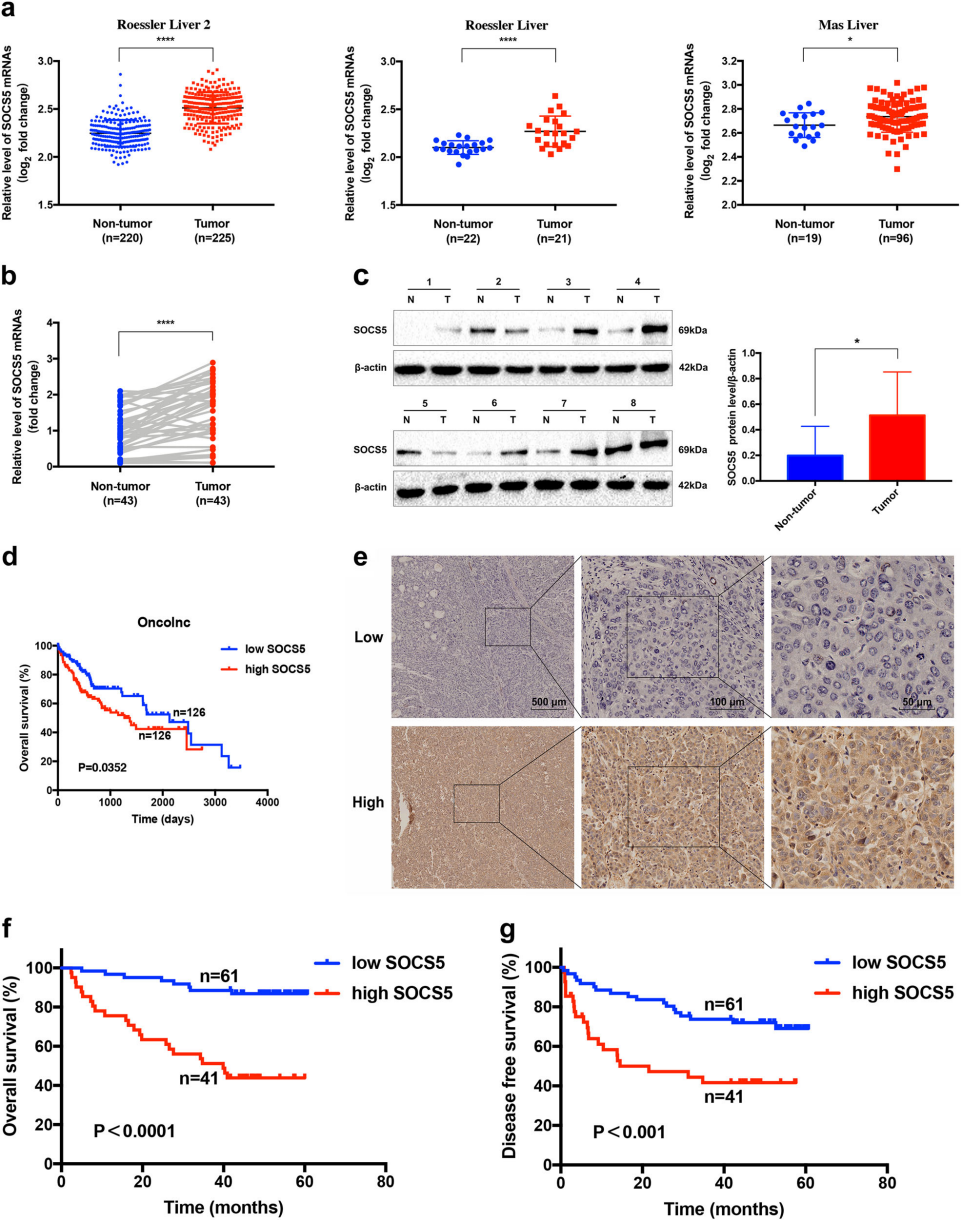
High suppressor of cytokine signaling 5 (SOCS5) expression predicts poor prognosis in hepatocellular carcinoma (HCC). **a** SOCS5 mRNA expression levels in HCC vs. non-tumor liver tissues were examined in three HCC datasets in the Oncomine database. **b** Quantitative PCR analysis of SOCS5 mRNA expression in HCC and non-tumor liver tissues in our patient cohort. SOCS5 mRNA expression levels were normalized according to glyceraldehyde 3-phosphate dehydrogenase expression levels (*n* = 43 per group). **c** Western blotting analysis of SOCS5 protein expression in HCC (T) and non-tumor liver tissues (N). SOCS5 protein expression levels were normalized according to β-actin expression levels (*n* = 8 per group). **d** Correlation of SOCS5 mRNA expression in HCC patients with overall survival (OS) rate in the Oncolnc database. The patients (*n* = 252) were stratified into two groups, and patients with high SOCS5 mRNA expression had much shorter OS than did patients with low SOCS5 mRNA expression. **e** Immunohistochemical analysis of SOCS5 protein expression in human HCC tissues. **f** Correlation of SOCS5 protein expression with OS rate in HCC patients. **g** Correlation of SOCS5 protein expression with disease-free survival rate in HCC patients. *P < 0.05, ****P < 0.0001

We retrieved the mRNA expression of SOCS5 from three published HCC datasets in the Oncomine database and observed significant overexpression of SOCS5 in HCC tissues compared to the non-tumor liver tissues in all the three datasets (Fig. 1a). We validated this observation in our patient cohort using qPCR and western blotting of the matched HCC and adjacent non-tumor frozen tissues and similarly observed significant overexpression of SOCS5 in HCC (Fig. 1b, c).

### High SOCS5 expression predicts poor prognosis in HCC

Using published datasets from the Oncolnc database, we found that HCC patients with high SOCS5 mRNA expression had significantly worse median overall survival (OS) than patients with low SOCS5 (OS: 1372 vs. 2131 days, respectively; Fig. 1d). To validate this at the protein level, the expression of SOCS5 in 102 paraffin-embedded HCC tissues was examined by IHC and correlated with clinicopathological characteristics of HCC patients (Table 1). Among these 102 HCC samples, 61 had low SOCS5 expression, and 41 had high SOCS5 expression (representative figures shown in Fig. 1e). High SOCS5 expression was positively correlated with malignant features such as high AFP level (*χ*^2^ = 3.972, *P* < 0.05), larger tumor size (*χ*^2^ = 7.135, *P* < 0.01), microvascular invasion (*χ*^2^ = 4.178, *P* < 0.05), portal vein invasion (*χ*^2^ = 4.374, *P* < 0.05) and higher tumor, node, metastasis (TNM) stage (*χ*^2^ = 12.270, *P* < 0.01) (Table 1). Kaplan–Meier analysis using our IHC data indicated that HCC patients with high SOCS5 expression had significantly shorter median OS and disease-free survival (DFS) than those with low SOCS5 expression (OS: 36.5 vs. 55.8 months, respectively; DFS: 29.4 vs. 48.3 months, respectively; Fig. 1f, g). Univariate and multivariate analyses further demonstrated that SOCS5 expression was negatively correlated with both OS and DFS (Tables 2, 3) and that high SOCS5 expression is an independent risk factor for HCC prognosis.

**Table 2:** Univariate and multivariate analysis of clinicopathological factors for OS

Variables	Univariate		Multivariate	
	HR (95% CI)	*P* value	HR (95% CI)	*P* value
Gender, male/female	1.071 (0.686–1.672)	0.764		
Age (years, <50 vs. ≥50)	2.757 (1.320–5.769)	**0.007**	3.073 (1.421–6.648)	**0.004**
Alcoholism, yes/no	2.270 (1.117–4.610)	**0.023**	2.428 (1.187–4.965)	**0.015**
Liver cirrhosis, yes/no	1.217 (0.544–2.721)	0.633		
AFP (ng/L, ≤400 vs. >400)	2.320 (1.142–4.713)	**0.020**		
ALT (U/L, ≤60 vs. >60)	1.199 (0.492–2.924)	0.689		
AST (U/L, ≤42 vs. >42)	1.276 (0.587–2.771)	0.538		
Tumor number, single/multiple	2.025 (0.905–4.532)	0.086		
Tumor size (cm, ≤5 vs. >5)	3.301 (1.624–6.707)	**0.001**		
Microvascular invasion, yes/no	2.563 (1.266–5.190)	**0.009**		
Portal vein invasion, yes/no	3.511 (1.341–9.196)	**0.011**	3.519 (1.274–9.719)	**0.015**
TNM stage (I vs. II vs. III)	2.487 (1.611–3.838)	**0.000**		
SOCS5 (low vs. high)	5.937 (2.648–13.310)	**0.000**	5.515 (2.437–12.480)	**0.000**

Bold numbers indicate significant differences (*P* < 0.05)

*OS* overall survival, *HR* hazard ratio, *CI* confidence interval, *AFP* α-Fetoprotein, *ALT* alanine aminotransferase, *AST* aspartate aminotransferase, *TNM* tumor, node, metastasis, *SOCS5* suppressor of cytokine signaling 5 ﻿

**Table 3:** Univariate and multivariate analysis of clinicopathological factors for DFS

Variables	Univariate		Multivariate	
	HR (95% CI)	*P* value	HR (95% CI)	*P* value
Gender, male/female	1.082 (0.478–2.448)	0.850		
Age (years, <50 vs. ≥50)	1.519 (0.723–3.195)	0.270		
Alcoholism, yes/no	1.417 (0.739–2.717)	0.294		
Liver cirrhosis, yes/no	1.426 (0.631–3.223)	0.394		
AFP (ng/L, ≤400 vs. >400)	1.670 (0.879–3.172)	0.117		
ALT (U/L, ≤60 vs. >60)	1.284 (0.641–2.572)	0.480		
AST (U/L, ≤42 vs. >42)	1.213 (0.617–2.386)	0.576		
Tumor number, single/multiple	2.346 (1.142–4.816)	**0.020**		
Tumor size (cm, ≤5 vs. >5)	2.062 (1.097–3.876)	**0.025**		
Microvascular invasion, yes/no	1.212 (0.624–2.351)	0.570		
Portal vein invasion, yes/no	1.877 (0.665–5.298)	0.234		
TNM stage (I vs. II vs. III)	1.710 (1.137–2.573)	**0.010**		
SOCS5 (low vs. high)	2.818 (1.501–5.290)	**0.001**	2.627 (1.392–4.960)	**0.003**

Bold numbers indicate significant differences (*P* < 0.05)

*DFS* disease-free survival, *HR* hazard ratio, *CI* confidence interval, *AFP* α-Fetoprotein, *ALT* alanine aminotransferase, *AST* aspartate aminotransferase, *TNM* tumor, node, metastasis, *SOCS5* suppressor of cytokine signaling 5 ﻿

### SOCS5 is a regulator of the PI3K/Akt/mTOR pathway

**Fig. 2:**
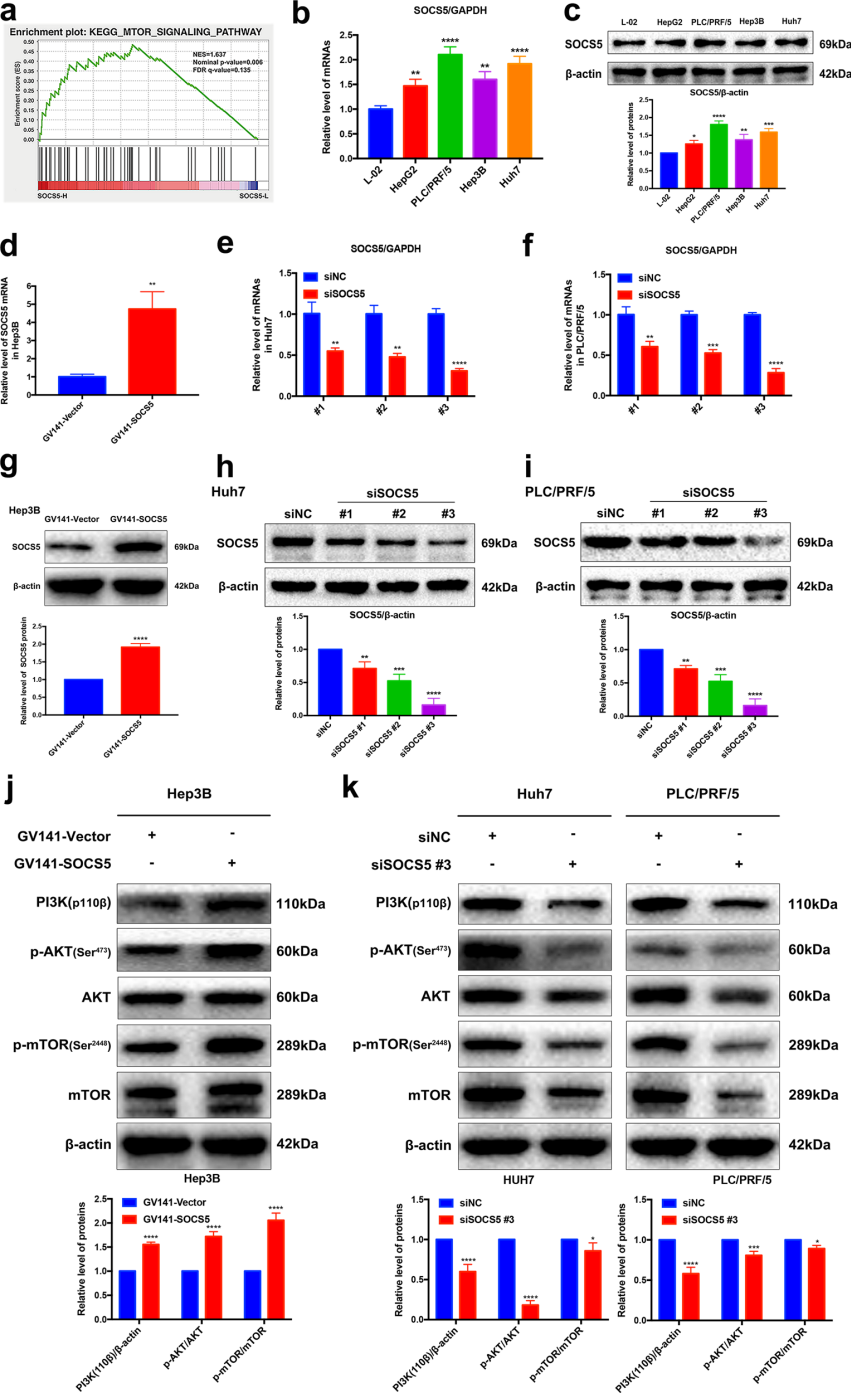
Suppressor of cytokine signaling 5 (SOCS5) is a regulator of the phosphoinositide-3 kinase (PI3K)/Akt/mammalian target of rapamycin (mTOR) pathway. **a** Gene Set Enrichment Analysis showing a significant positive correlation between SOCS5 expression and the MTOR_SIGNALING pathway in hepatocellular carcinoma. **b** Quantitative PCR (qPCR) analysis of SOCS5 basal mRNA expression in five cell lines. SOCS5 mRNA expression levels were normalized according to the glyceraldehyde 3-phosphate dehydrogenase (GAPDH) expression levels. **c** Western blotting analysis of SOCS5 basal protein expression in the five cell lines; β-actin was used as a loading control. **d** qPCR analysis of SOCS5 mRNA expression in Hep3B cells transfected with GV141-Vector and GV141-SOCS5 for 24 h. SOCS5 mRNA expression levels were normalized according to the GAPDH expression levels. **e**, **f** qPCR analysis of SOCS5 mRNA expression in Huh7 (**e**) and PLC/PRF/5 (**f**) cells transfected with siSOCS5 #1, #2, #3 and negative control siNC for 24 h. SOCS5 mRNA expression levels were normalized according to the GAPDH expression levels. **g** Western blotting analysis of SOCS5 protein expression in Hep3B cells transfected with GV141-Vector and GV141-SOCS5 for 24 h. β-Actin was used as a loading control. **h**, **i** Western blotting analysis of SOCS5 protein expression in Huh7 (**h**) and PLC/PRF/5 (**i**) cells transfected with siSOCS5 #1, #2, or #3 and siNC for 24 h. β-Actin was used as a loading control. **j** Western blotting analysis of the PI3K 110β, p-AKT, AKT, p-mTOR, and mTOR protein expression in Hep3B cells transfected with GV141-Vector and GV141-SOCS5 for 24 h. β-Actin was used as a loading control. **k** Western blotting analysis of PI3K 110β, p-AKT, AKT, p-mTOR and mTOR protein expression in Huh7 and PLC/PRF/5 cells transfected with siNC and siSOCS5 #3 for 24 h. β-Actin was used as a loading control. Data are presented as mean ± S.D. from three independent experiments. *P < 0.05, **P < 0.01, ***P < 0.001, ****P < 0.0001

GSEA results demonstrated a significant positive correlation between SOCS5 expression in HCC and the MTOR_SIGNALING pathway (Fig. 2a). We further assessed the impact of SOCS5 on PI3K/Akt/mTOR pathway in Hep3B, which is the HCC cell line with low endogenous SOCS5 expression (Fig. 2b, c). Cells were transfected with GV141-SOCS5 to increase the SOCS5 expression, and great overexpression efficiency was observed (Fig. 2d, g). Subsequently, we assessed the impact of SOCS5 on PI3K/Akt/mTOR pathway in Huh7 and PLC/PRF/5, which are two HCC cell lines with high endogenous SOCS5 expression (Fig. 2b, c). Cells were transfected with three siRNAs specifically targeting SOCS5 (siRNA #1, #2, and #3) to knock down the SOCS5 expression. Greatest knockdown efficiency was observed with siRNA #3 (Fig. 2e, f, h, i); therefore, siRNA #3 was used in all subsequent studies.

Western blotting showed that the expression levels of PI3K 110β, phospho-Akt (p-Akt), and phospho-mTOR (p-mTOR) levels increased in Hep3B cells at 24 h after SOCS5 overexpression (Fig. 2j). Conversely, following SOCS5 knockdown with siSOCS5 #3 for 24 h, the expression levels of PI3K 110β, p-Akt, and p-mTOR were markedly decreased in Huh7 and PLC/PRF/5 cells (Fig. 2k). Moreover, our further research revealed that SOCS5 overexpression and knockdown upregulated and downregulated the expression levels of phospho-ULK1 (p-ULK1), respectively, and that SOCS5 overexpression and knockdown can downregulate and upregulate the expression levels of phospho-ATG13 (p-ATG13), respectively (Supplementary Fig. S1). These data suggest that SOCS5 is a regulator of the PI3K/Akt/mTOR pathway, as predicted by our GSEA data.

### SOCS5 expression levels affect HCC cell autophagy

**Fig. 3:**
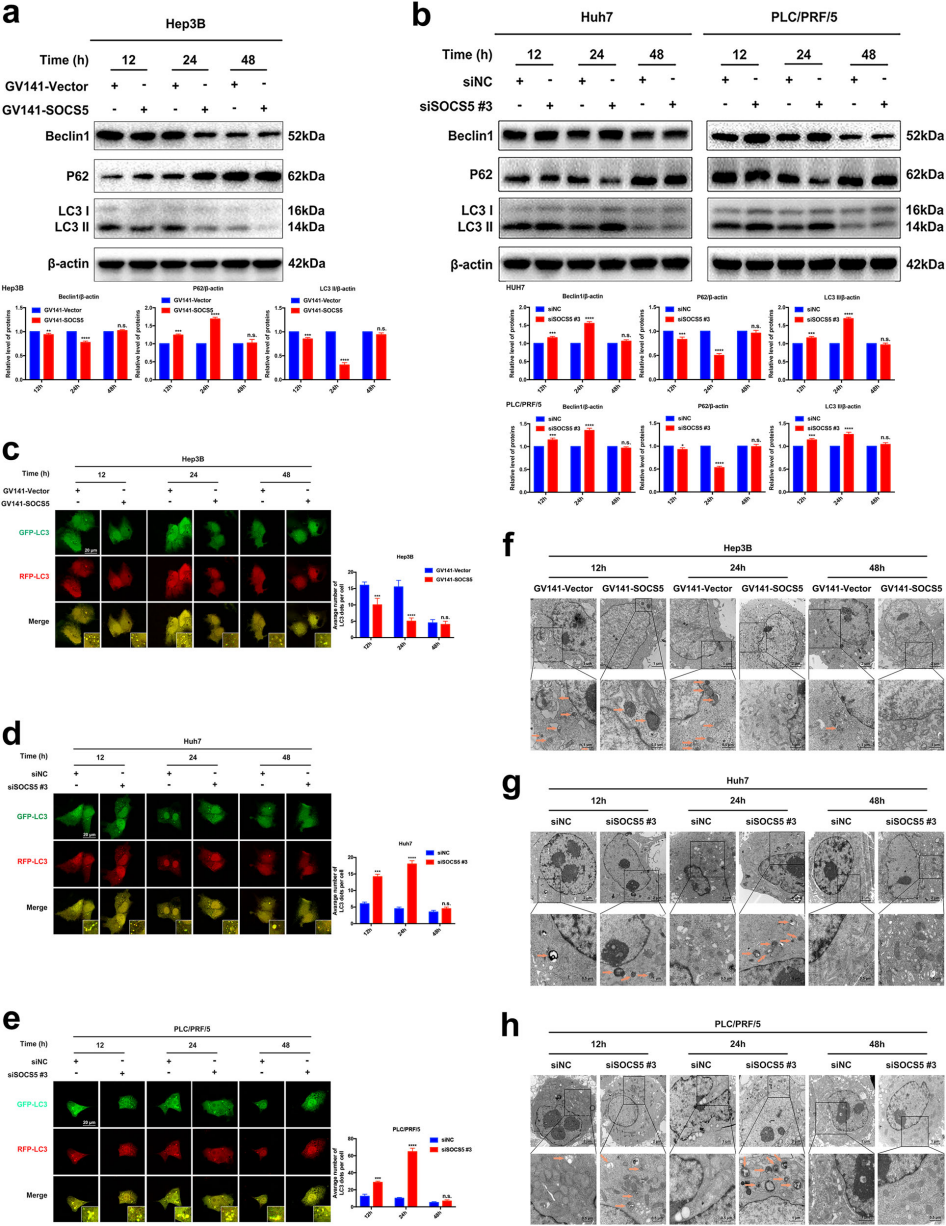
Suppressor of cytokine signaling 5 (SOCS5) expression levels affects hepatocellular carcinoma cell autophagy. **a** Western blotting analysis of Beclin1, P62, and LC3-II protein expression in Hep3B cells transfected with GV141-Vector and GV141-SOCS5 for 12, 24, and 48 h. β-Actin was used as a loading control. **b** Western blotting analysis of Beclin1, P62, and LC3-II protein expression in Huh7 and PLC/PRF/5 cells transfected with siNC and siSOCS5 #3 for 12, 24, and 48 h. β-Actin was used as a loading control. **c** Immunofluorescence images of Hep3B cells transfected with GV141-Vector/GV141-SOCS5 and GFP-RFP-LC3 for 12, 24, and 48 h. **d**, **e** Immunofluorescence images of Huh7 (**d**) and PLC/PRF/5 (**e**) cells transfected with siNC/siSOCS5 #3 and GFP-RFP-LC3 for 12, 24, and 48 h. **f** Representative electron micrographs of autophagic vesicles in Hep3B cells transfected with GV141-Vector and GV141-SOCS5 for 12, 24, and 48 h. **g**, **h** Representative electron micrographs of autophagic vesicles in Huh7 (**g**) and PLC/PRF/5 (**h**) cells transfected with siNC and siSOCS5 #3 for 12, 24, and 48 h. Data are presented as mean ± S.D. from three independent experiments. *P < 0.05, **P < 0.01, ***P < 0.001, ****P < 0.0001

Since SOCS5 can regulate the PI3K/Akt/mTOR pathway and then affect p-ULK1/p-ATG13, which play an important role in affecting autophagy^19–21^, we further examined the relationship between SOCS5 and autophagy-related protein expression in HCC cells that were transfected with GV141-SOCS5 or siSOCS5 #3 for 12, 24, or 48 h. We found that the expression levels of Beclin 1 and LC3II decreased most markedly after SOCS5 was overexpressed for 24 h, while P62 protein expression was significantly increased (Fig. 3a). Conversely, the expression levels of Beclin 1 and LC3II increased most markedly after SOCS5 was inhibited for 24 h, while P62 protein expression was significantly reduced (Fig. 3b). Moreover, when visualized by immunofluorescence, we observed that the number of LC3 dots was significantly decreased in Hep3B cells transfected with GV141-SOCS5 for 24 h (Fig. 3c), and the number of LC3 dots was significantly increased in both Huh7 and PLC/PRF/5 cells transfected with siSOCS5#3 for 24 h (Fig. 3d, e). Interestingly, the number of autophagic vesicles was also significantly decreased in Hep3B cells transfected with GV141-SOCS5 for 24 h (Fig. 3f), and the number of autophagic vesicles was observed to be greatest in Huh7 and PLC/PRF/5 cells transfected with siSOCS5#3 for 24 h (Fig. 3g, h). Moreover, we performed an autophagy flux assay using Baf A1 (lysosomal ATPase inhibitor) and observed that SOCS5 overexpression/knockdown for 24 h led to the decrease/increase in LC3-II protein expression, and LC3-II protein expression levels were further increased by Baf A1 (Supplementary Fig S2). These data suggest that SOCS5 overexpression/knockdown can decrease/increase formation of autophagosomes upstream of the lysosomes. Collectively, our data demonstrated that SOCS5 overexpression inhibited autophagy and SOCS5 inhibition induced autophagy in HCC cells. Since the inhibition/induction of autophagy by SOCS5 overexpression/inhibition was most pronounced 24 h after transfection in HCC cells, we selected the 24 h time point for all subsequent studies.

### Effects of SOCS5 expression levels on HCC cell migration and invasion via autophagy mediation

**Fig. 4:**
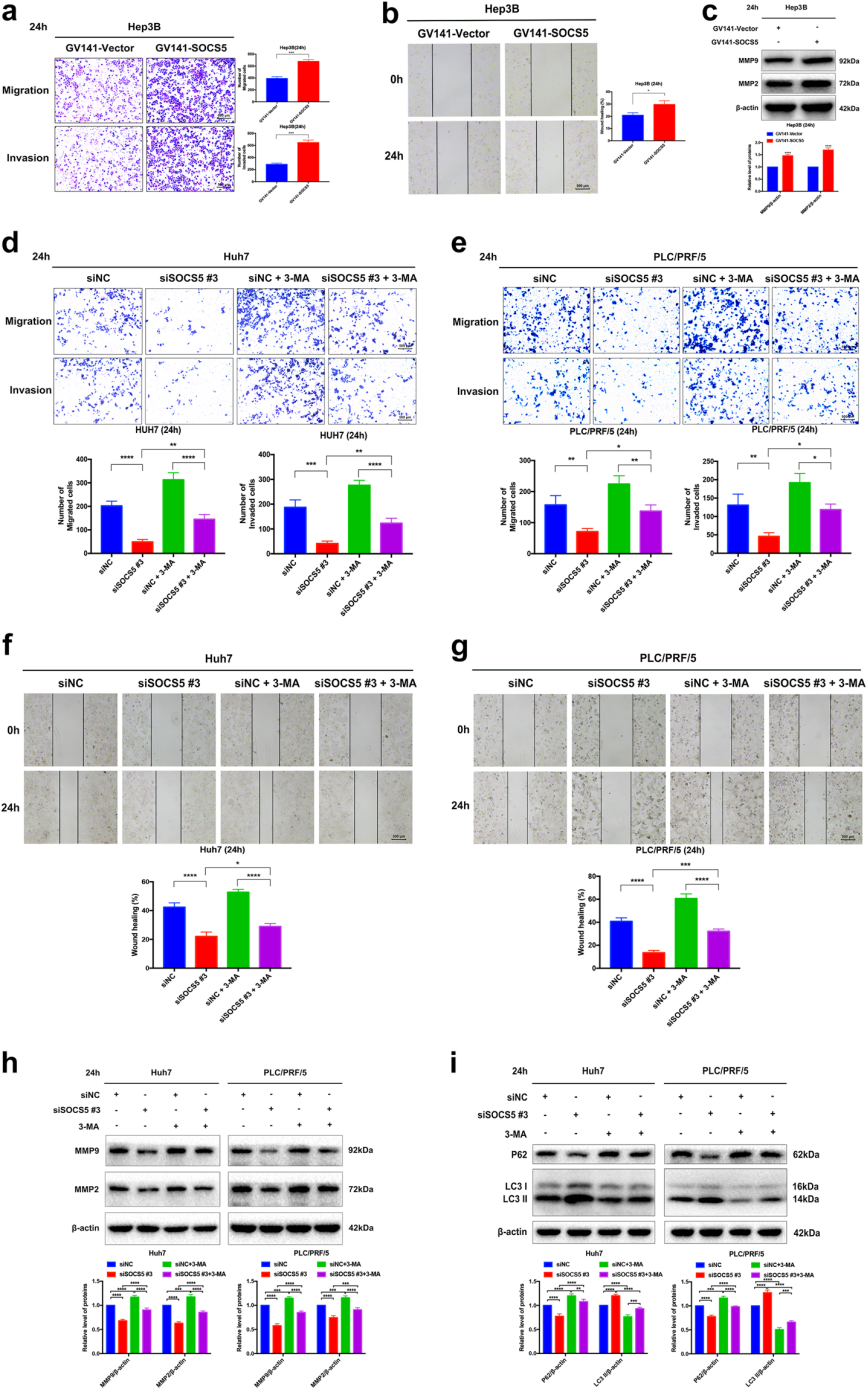
Effects of suppressor of cytokine signaling 5 (SOCS5) expression levels on hepatocellular carcinoma cell migration and invasion via autophagy mediation. **a**–**c** Hep3B cells were transfected with GV141-Vector and GV141-SOCS5 for 24 h. **a** Comparison of the migration and invasion Hep3B cells using transwell compartments. **b** Wound-healing assay comparing the motility of Hep3B cells. The wound-healing area was analyzed using the ImageJ software. **c** Western blotting analysis of matrix metalloproteinase 9 (MMP9) and MMP2 protein expression. β-Actin was used as a loading control. **d**–**i** Huh7 and PLC/PRF/5 cells were transfected with siNC and siSOCS5 #3 or treated with phosphate-buffered saline (control) or 3-methyladenine (2 mM) or a combination of both treatments for 24 h. **d**, **e** Comparison of the migration and invasion of Huh7 (**d**) and PLC/PRF/5 (**e**) cells using transwell compartments. **f**, **g** Wound-healing assay comparing the motility of Huh7 (**f**) and PLC/PRF/5 (**g**) cells. The wound-healing area was analyzed using the ImageJ software. **h** Western blotting analysis of MMP9 and MMP2 protein expression. β-Actin was used as a loading control. **i** Western blotting analysis of P62 and LC3-II protein expression. β-Actin was used as a loading control. Data are presented as mean ± S.D. from three independent experiments. *P < 0.05, **P < 0.01, ***P < 0.001, ****P < 0.0001

The induction of autophagy has been reported to contribute toward cancer cell migration and invasion^18^. We therefore examined the effect of inhibited/induced autophagy caused by SOCS5 overexpression/inhibition on HCC cell migration and invasion. We observed that SOCS5 overexpression with GV141-SOCS5 caused significantly increased cell migration and invasion in Hep3B cell line (Fig. 4a, b), consistent with increased matrix metalloproteinase 9 (MMP9) and MMP2 protein expression (Fig. 4c). Conversely, SOCS5 suppression with siSOCS5 #3 caused significantly reduced cell migration and invasion (Fig. 4d–g) in Huh7 and PLC/PRF/5 cell lines, consistent with decreased MMP9 and MMP2 protein expression (Fig. 4h). Moreover, SOCS5 suppression-mediated anti-metastasis effects were reversed when cells were co-treated with siSOCS5 #3 and the autophagy inhibitor 3-MA (Fig. 4d–h). We also confirmed that co-treatment with 3-MA significantly decreased SOCS5 inhibition-dependent LC3-II protein expression (Fig. 4i). Thus our results demonstrated that SOCS5 overexpression promoted HCC cell migration and invasion via autophagy inhibition. However, SOCS5 suppression induced autophagy, resulting in inhibition of HCC cell migration and invasion.

### Dual inhibition of SOCS5 and mTOR enhances autophagy and exerts cooperative anti-metastatic effects on HCC cells

**Fig. 5:**
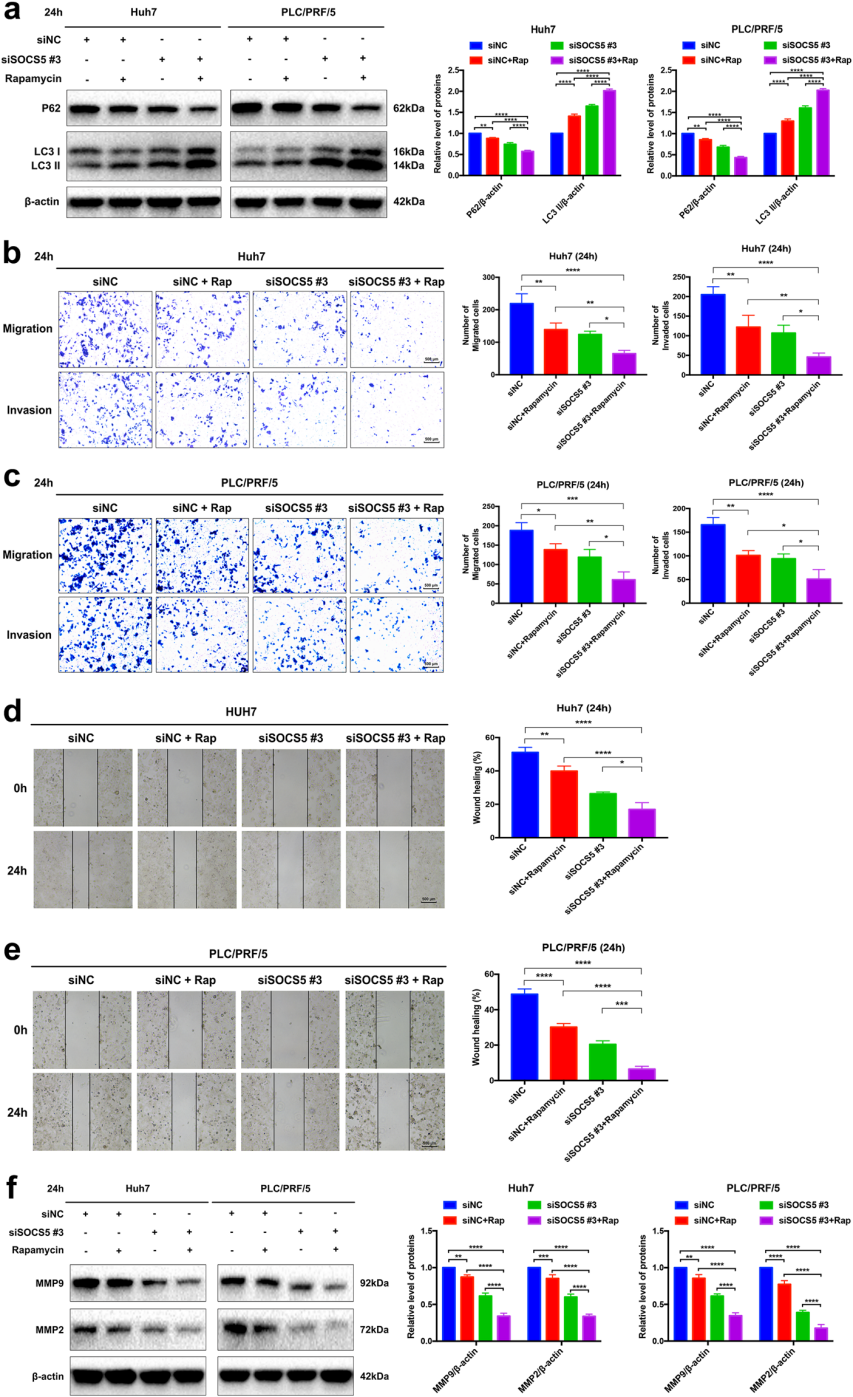
Dual inhibition of suppressor of cytokine signaling 5 (SOCS5) and mammalian target of rapamycin enhances autophagy and exerts cooperative anti-metastatic effects on hepatocellular carcinoma cells. **a**–**f** Huh7 and PLC/PRF/5 cells were treated with rapamycin (100 nM) or dimethyl sulfoxide or transfected with siNC and siSOCS5 #3 or a combination of both treatments for 24 h. **a** Western blotting analysis of P62 and LC3-II protein expression. β-Actin was used as a loading control. **b**, **c** Comparison of the migration and invasion of Huh7 (**b**) and PLC/PRF/5 (**c**) cells using transwell compartments. **d**, **e** Wound-healing assay comparing the motility of Huh7 (**d**) and PLC/PRF/5 (**e**) cells. The wound-healing area was analyzed using the ImageJ software. **f** Western blotting analysis of matrix metalloproteinase 9 (MMP9) and MMP2 protein expression. β-Actin was used as a loading control. Data are presented as mean ± S.D. from three independent experiments. *P < 0.05, **P < 0.01, ***P < 0.001, ****P < 0.0001

Given our observation of the relationship between SOCS5, the mTOR pathway, and autophagy, we next investigated whether the combined targeting of SOCS5 and mTOR may lead to enhanced effects on HCC cell migration and invasion. We observed that rapamycin (mTOR inhibitor) alone or SOCS5 suppression alone increased LC3-II protein expression while reducing P62 protein expression compared to that of the control and that combination of both showed an enhanced effect (Fig. 5a). Similar to SOCS5 suppression, mTOR inhibition by rapamycin alone also caused a reduction in HCC cell migration and invasion with concomitant reduction of the protein expression of MMP9 and MMP2 (Fig. 5b–f). The combination of rapamycin and SOCS5 suppression led to a significantly enhanced suppression of migration and invasion (Fig. 5b–f). Taken together, our results demonstrated that combined suppression of SOCS5 and mTOR significantly increased autophagic activity, leading to cooperative anti-metastatic effects in Huh7 and PLC/PRF/5 cells.

### SOCS5 knockdown inhibits HCC cells metastasis in vivo

**Fig. 6:**
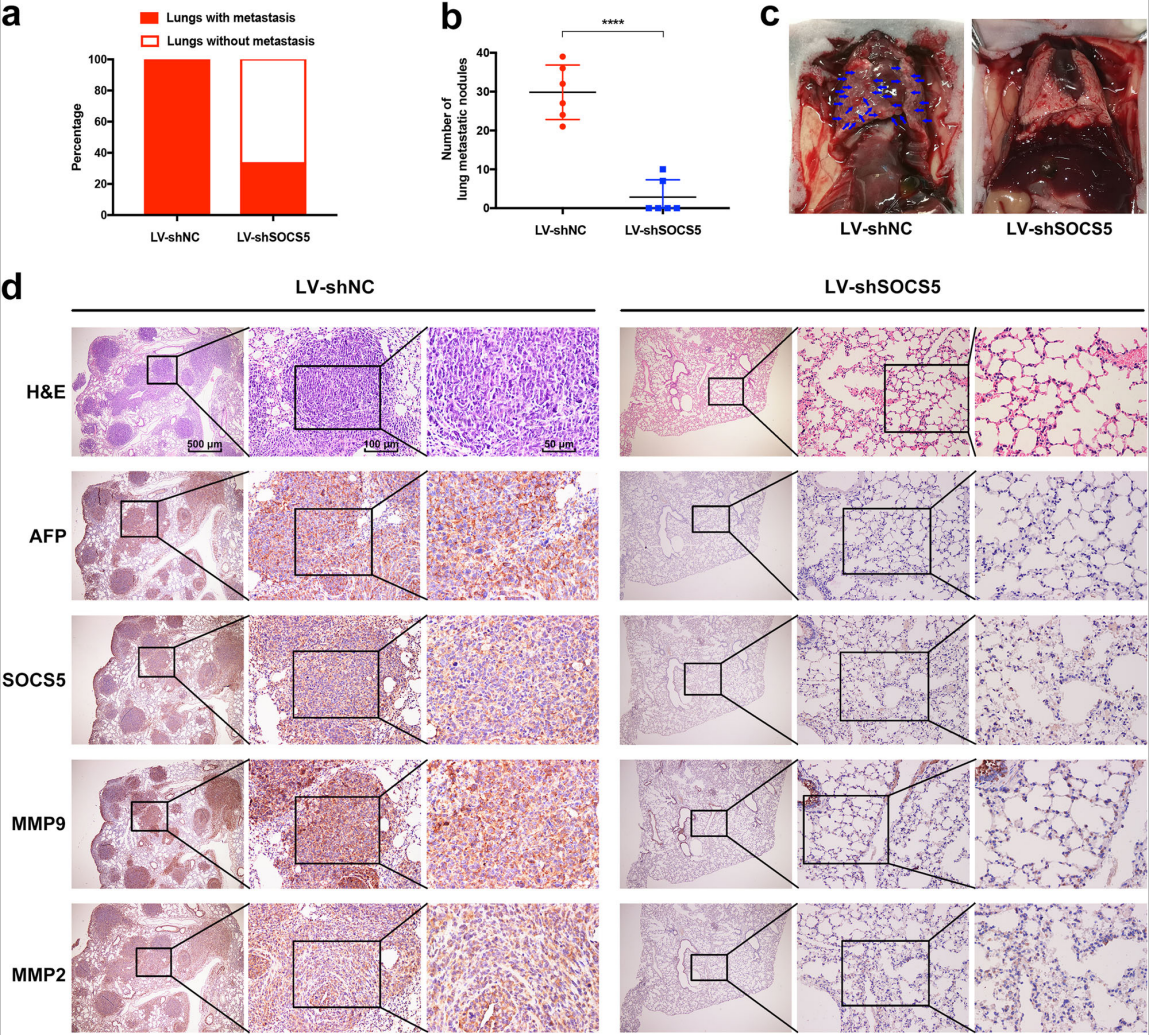
Suppressor of cytokine signaling 5 (SOCS5) knockdown inhibits hepatocellular carcinoma cells’ metastasis in vivo. Briefly, 2 × 10^6^ Huh7^LV-shNC^ cells or 2 × 10^6^ Huh7^LV-shSOCS5^ cells suspended in 200 μL phosphate-buffered saline were injected into the tail veins of nude mice (*n* = 6 per group). **a** Lung metastasis rate from two groups. **b** The number of metastatic nodules in the lungs from two groups. Data are presented as mean ± SD. **c** Representative images of lung tissues from nude mice. **d** Hematoxylin and eosin staining and immunohistochemical staining of α-fetoprotein, SOCS5, matrix metalloproteinase 9 (MMP9), and MMP2 protein expression in the lungs. ****P < 0.0001

We further examined the in vivo anti-metastasis effects of SOCS5 inhibition in the lateral tail vein metastasis model (lentivirus infection rate as shown in Supplementary Fig. S3). Compared with nude mice inoculated with negative control Huh7^LV-shNC^ cells, the lung metastasis rate and the number of metastatic nodules in nude mice inoculated with Huh7^LV-shSOCS5^ cells were significantly reduced (Fig. 6a–c), which indicated that the metastatic ability of Huh7 cells was significantly reduced by shRNA of SOCS5. Importantly, IHC staining data, which showed positive AFP staining in these metastatic nodules (Fig. 6d), further validated that these nodules were indeed derived from HCC cells. Moreover, IHC was also used to evaluate the expression of SOCS5, MMP9, and MMP2 in the lungs. We observed that the SOCS5, MMP9, and MMP2 expression in the Huh7^LV-shSOCS5^ group decreased more significantly than that in the Huh7^LV-shNC^ group (Fig. 6d). Our in vivo data confirmed the in vitro results that SOCS5 inhibition exerts anti-metastatic effects.

## Discussion

**Fig. 7:**
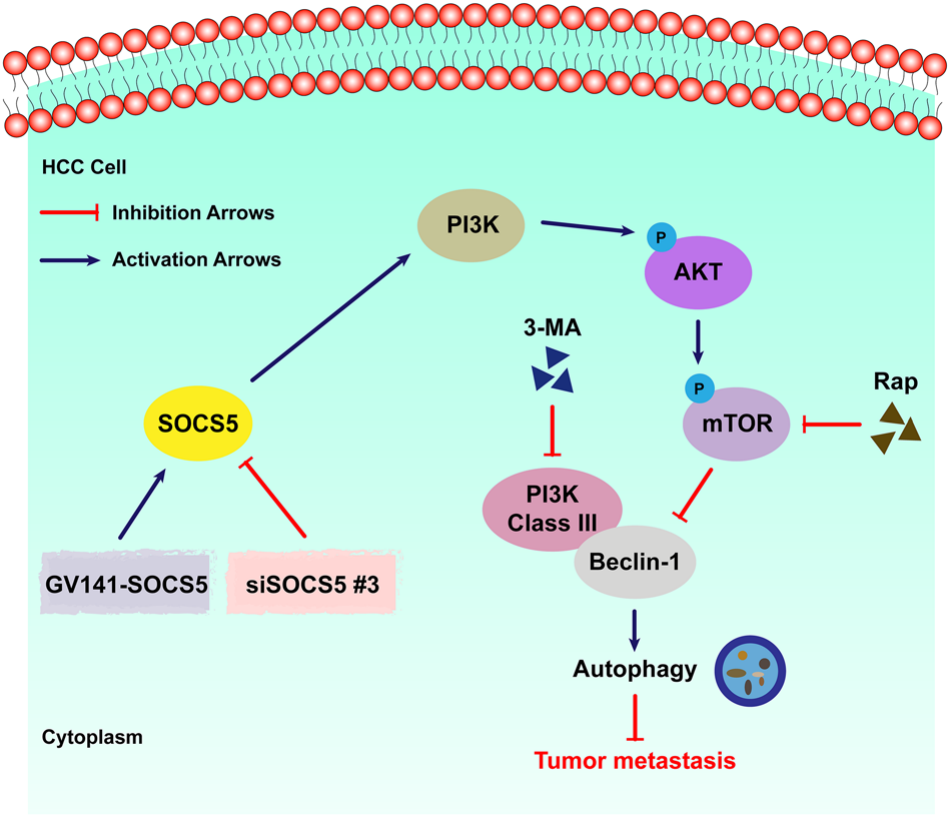
**Schematic diagram depicting the mechanism by which suppressor of cytokine signaling 5 promotes or inhibits hepatocellular carcinoma cell metastasis via phosphoinositide-3 kinase/Akt/mammalian target of rapamycin pathway-mediated autophagy**

Using large independent datasets and our own patient cohort, we confirmed the significantly enhanced expression of SOCS5 in HCC tissues compared to non-tumor liver tissues. Clinically, the overexpression of SOCS5 in HCC patients was significantly correlated with aggressive tumor features such as increased AFP level, large tumor size, vascular invasion, and advanced TNM stage. Consistently, SOCS5 expression level in HCC is an independent predictor of the OS and DFS of HCC patients; HCC patients with higher SOCS5 expression have poorer prognosis than those with lower SOCS5 expression. Functionally, we uncovered a novel mechanism of SOCS5 in regulating autophagy at least in part via the PI3K/Akt/mTOR pathway, which in turn regulated HCC cell migration, invasion, and metastasis. Dual inhibition of SOCS5 and mTOR enhanced autophagy and exerted cooperative anti-metastatic effects on HCC cells, as illustrated in the schematic diagram in Fig. 7. Our in vitro and in vivo data collectively support that SOCS5 inhibition has anti-metastatic effects and correlated with our clinical observations that high SOCS5 expression is associated with more aggressive features and poorer survival.

Our large-scale study on SOCS5 expression in HCC validated the observation from a previous report, in which only three pairs of HCC samples were studied^7^. Our clinical and functional studies suggest that SOCS5 plays an important role in promoting HCC progression, in particular by enhancing cell metastasis. This is opposed to a recent report suggesting a tumor-suppressive role of SOCS5 in HCC, whereby SOCS5 inhibition was observed to enhance anchorage-dependent and anchorage-independent colony growth of SNU398 and HepG2 cells^12^. However, these authors had not examined SOCS5 expression in HCC samples and studied cell growth as the only phenotype. In vivo, our lateral tail vein metastasis model generated from HCC cells with stable knockdown of SOCS5 showed decreased metastatic ability, whereas Sanchez-Mejias et al. showed increased growth of their xenografts from HCC cells transfected with seemingly transient SOSC5 siRNA. Thus the discrepancy of our conclusions may result from the differences in our assays; it is also possible that SOCS5 regulates HCC growth and progression in a complex manner, perhaps through the modulation of autophagy, which has been shown to be a double-edged sword in cancers, including HCC^13^.

Our observed correlation of SOCS5 expression with the mTOR pathway (from GSEA analysis) led us to investigate the novel role of SOCS5 in autophagy, a process which is modulated by the PI3K/Akt/mTOR pathway. Specifically, our data demonstrated that SOCS5 overexpression upregulated the PI3K/Akt/mTOR pathway, resulting in inhibition of autophagy and promotion of HCC cell migration and invasion in vitro. And SOCS5 inhibition downregulated the PI3K/Akt/mTOR pathway, resulting in induction of autophagy and inhibition of HCC cell migration and invasion in vitro and inhibition of HCC cell metastasis in vivo. The autophagy inhibitor, 3-MA, partially reversed the effects of SOCS5 inhibition on HCC cell migration and invasion, further confirming the contribution of autophagy toward these processes. This is consistent with the effects observed by Wang et al.: AFP activated PI3K/Akt/mTOR pathway to inhibit autophagy and promote metastasis^14^. Furthermore, the SOCS Box-containing protein-10 gene was reported to play a role in the autophagic lysosomal pathway^22^, again supporting our results that SOCS5 may play an important role in HCC cell autophagy. The loss of autophagy function was reported to be an essential condition for tumorigenesis^23^, consistent with our in vitro data showing that HCC cells with suppressed SOCS5 expression metastasize slower than the parental HCC cells with high SOCS5 expression.

Previous studies have demonstrated that strategies involving combined targeting of autophagy and another form of therapy can usually achieve better efficacy than monotherapy alone^24,25^. To this end, we have shown that the combined inhibition of mTOR (with rapamycin) and of SOCS5 (by RNA interference) achieved greatly enhanced autophagic effects compared to single inhibition of mTOR or SOCS5 alone. This enhanced autophagic effect resulted in greatly reduced HCC cell migration and invasion, as well as dramatically decreased MMP9 and MMP2 protein levels compared to either treatment alone. Thus co-targeting of mTOR and SOCS5 is a potential strategy that can significantly impair HCC cell metastasis.

In conclusion, we demonstrated that SOCS5 is overexpressed in HCC patients and that high SOCS5 expression is associated with aggressive tumor features and poorer patient prognosis. Functionally, we discovered a new role of SOCS5 in regulating PI3K/Akt/mTOR-mediated autophagy and the downstream processes of cell migration and invasion. The combined targeting of SOCS5 and the PI3K/Akt/mTOR pathway provides an attractive new option for impairing tumor metastasis, which is one of the main causes underlying the dismal prognosis of HCC patients.

## Supplementary information

Supplementary Figure S1

Supplementary Figure S2

Supplementary Figure S3

Supplementary Figure Legends

Supplementary Table 1

## Data Availability

All data generated or analyzed during this study are included in this published article or its Supplementary materials. Further details are available from the corresponding author upon reasonable request.
