# Correction: SOCS5 inhibition induces autophagy to impair metastasis in hepatocellular carcinoma cells via the PI3K/Akt/mTOR pathway

**DOI:** 10.1038/s41419-019-2009-z

**Published:** 2019-10-22

**Authors:** Mao Zhang, Shihai Liu, Mei-Sze Chua, Haoran Li, Dingan Luo, Sheng Wang, Shun Zhang, Chuandong Sun, Bing Han

**Affiliations:** 1 Department of Hepatobiliary and Pancreatic Surgery, Affiliated Hospital of Qingdao University, Qingdao, Shandong P. R. China; 2 Medical Animal Laboratory, Affiliated Hospital of Qingdao University, Qingdao, Shandong P. R. China; 3 Asian Liver Center, Department of Surgery, Stanford University School of Medicine, Stanford, CA USA; 4 Department of Colorectal Surgery, Fudan University Shanghai Cancer Center, Shanghai, P. R. China

**Keywords:** Metastasis, Metastasis

**Correction to: Cell Death & Disease**

10.1038/s41419-019-1856-y, published online 13 August 2019

Following publication of this article [1], it was realized that an error was made in typesetting which resulted in a change in the ordering of the last two authors. The correct ordering of the authors for the paper is as follows:

Mao Zhang, Shihai Liu, Mei-Sze Chua, Haoran Li, Dingan Luo, Sheng Wang, Shun Zhang, Chuandong Sun (*) & Bing Han (*)

[(*) indicates corresponding author]

We apologize for any inconvenience this may have caused the readers.

