# Supplementary figures and images for: SOCS5 inhibition induces autophagy to impair metastasis in hepatocellular carcinoma cells via the PI3K/Akt/mTOR pathway
Source: Cell Death Dis. 2019 Aug 13;10(8):612. doi: 10.1038/s41419-019-1856-y (PMC6690952; doi:10.1038/s41419-019-1856-y)

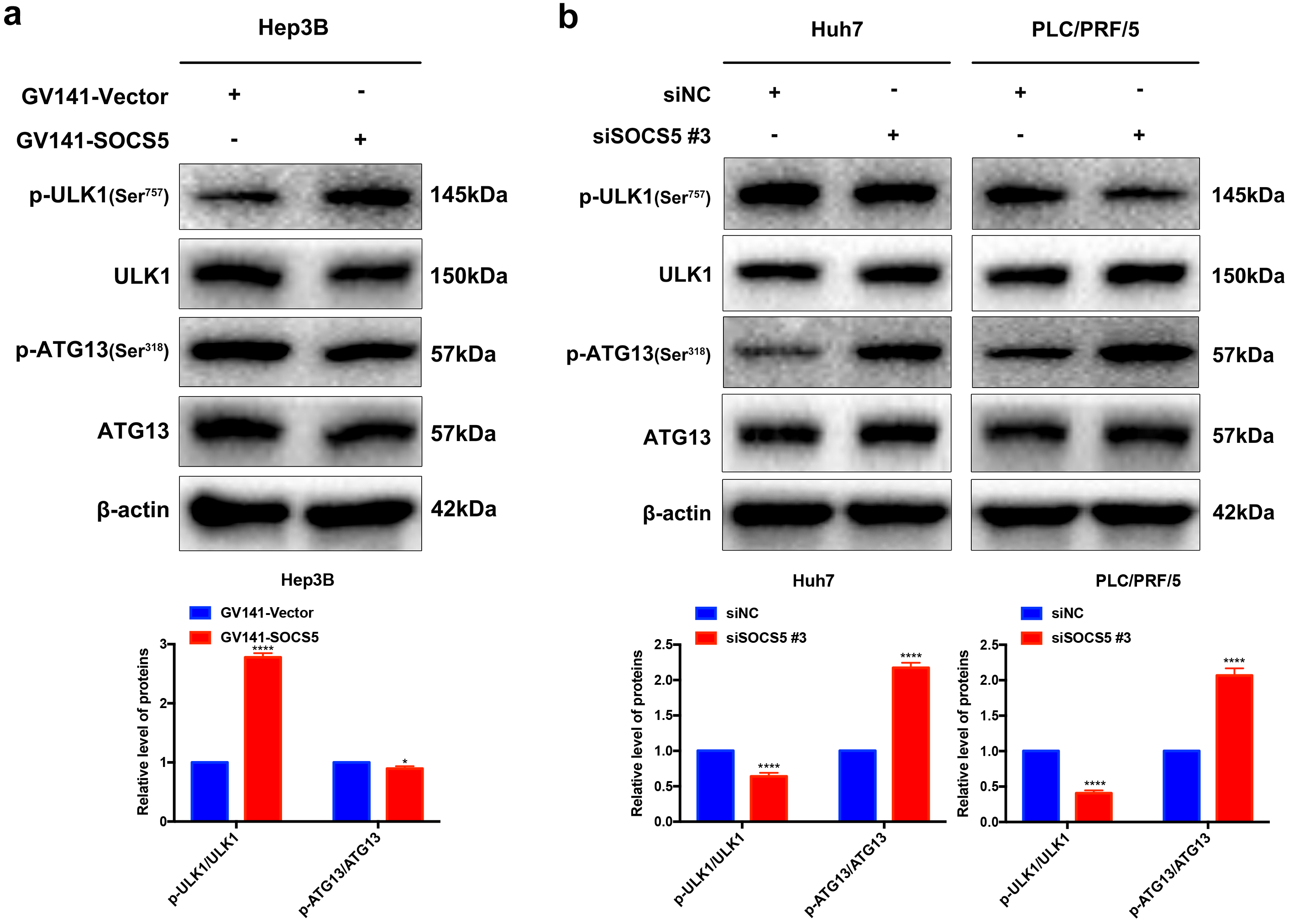

Supplement: Supplementary file 1 — Supplementary Figure S1 [file 41419_2019_1856_MOESM1_ESM.tif]

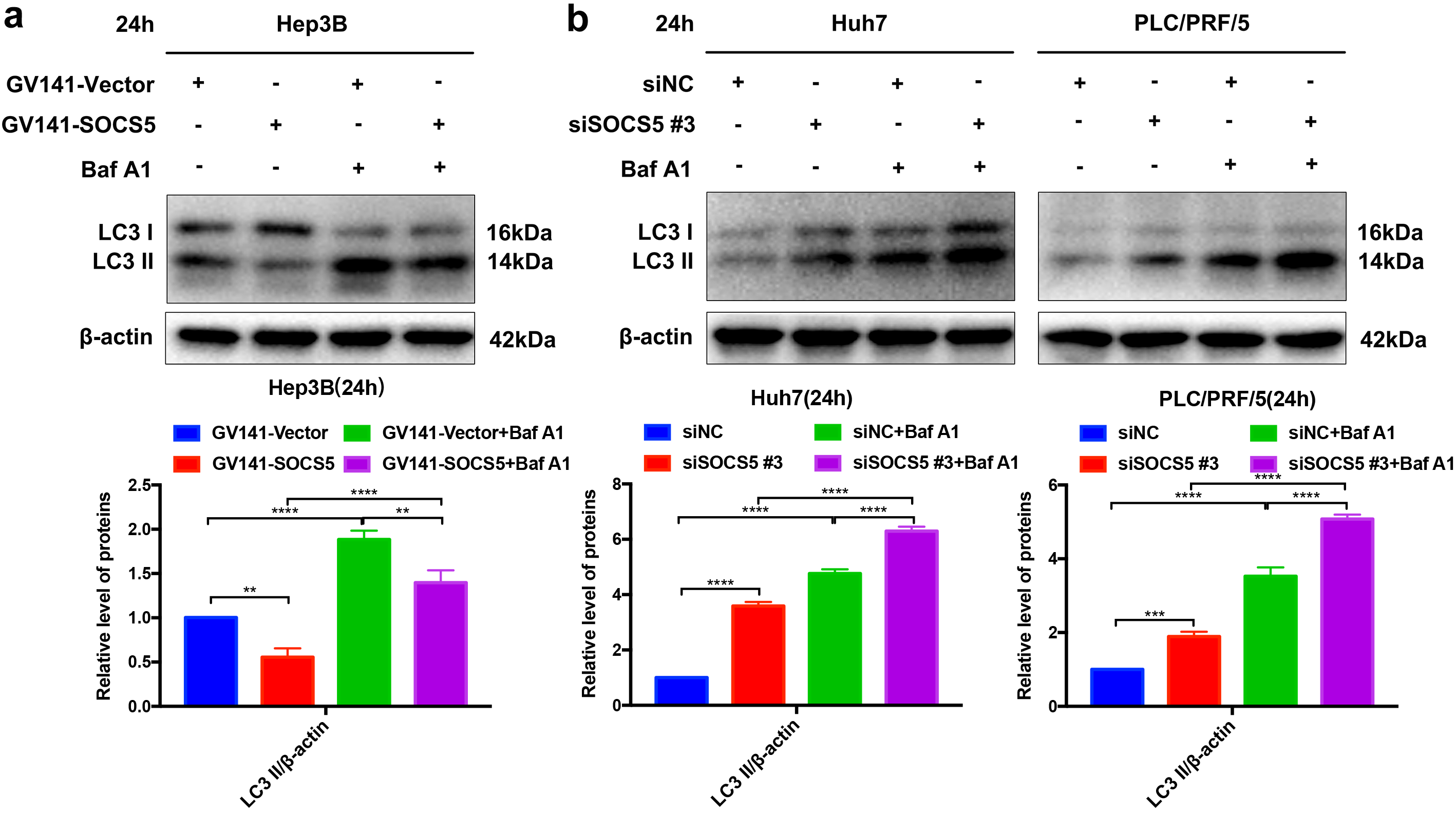

Supplement: Supplementary file 2 — Supplementary Figure S2 [file 41419_2019_1856_MOESM2_ESM.tif]

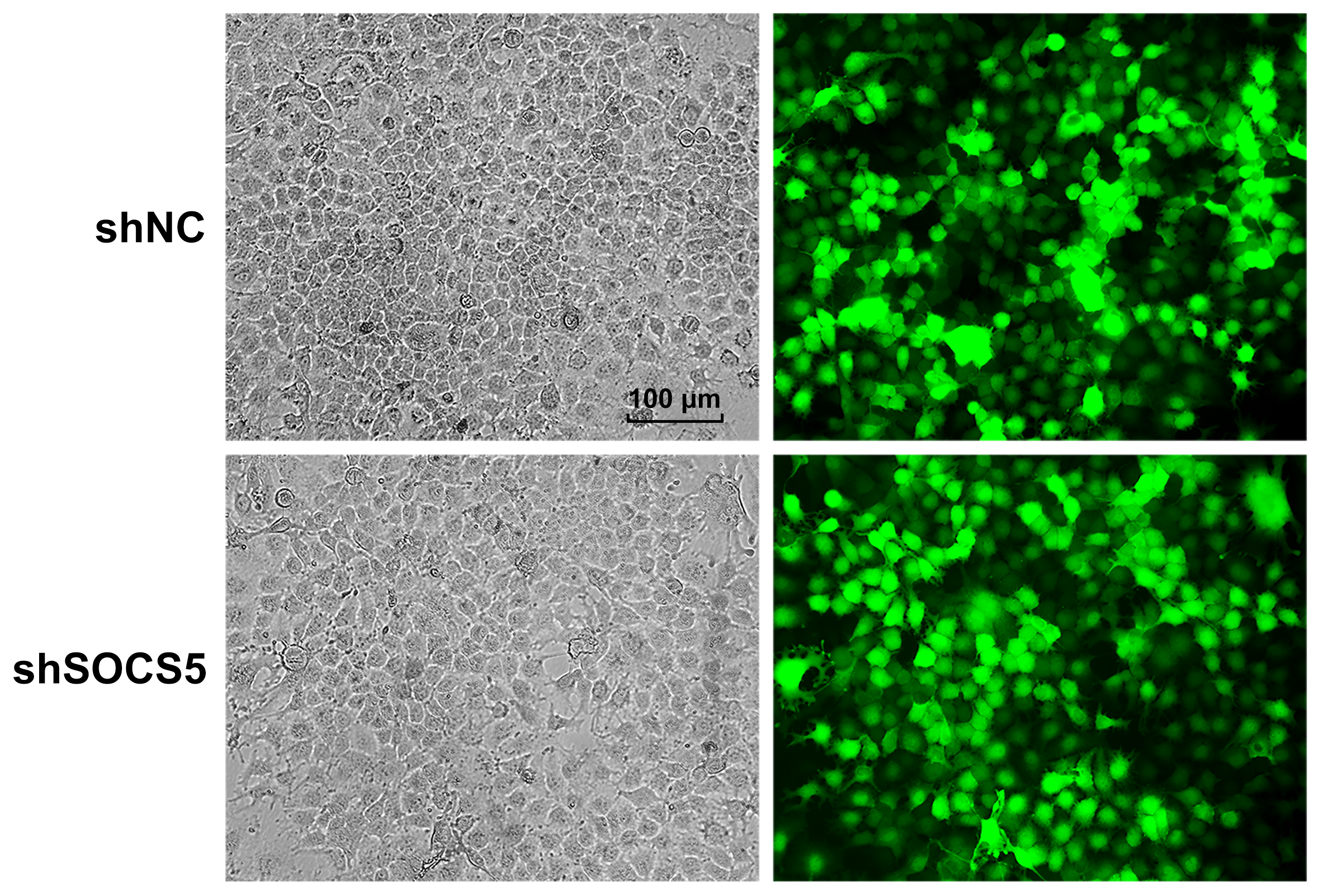

Supplement: Supplementary file 3 — Supplementary Figure S3 [file 41419_2019_1856_MOESM3_ESM.tif]
