# Supplementary material for: SOCS5 inhibition induces autophagy to impair metastasis in hepatocellular carcinoma cells via the PI3K/Akt/mTOR pathway
Source: Cell Death Dis. 2019 Aug 13;10(8):612. doi: 10.1038/s41419-019-1856-y (PMC6690952; doi:10.1038/s41419-019-1856-y)
Supplement: Supplementary file 4 — Supplementary Figure Legends [file 41419_2019_1856_MOESM4_ESM.docx]

**Supplementary Figure Legends**

**Supplementary Figure. S1. SOCS5 regulates p-ULK1 and p-ATG13. a-b.** HCC cells were transfected with GV141-Vector and GV141-SOCS5, or transfected with siNC and siSOCS5 #3 for 24 h. **a.** Western blotting analysis of p-ULK1, ULK1, p-ATG13 and ATG13 protein expression in Hep3B cells. β-actin was used as a loading control. **b.** Western blotting analysis of p-ULK1, ULK1, p-ATG13 and ATG13 protein expression in Huh7 and PLC/PRF/5 cells. β-actin was used as a loading control. The data are presented as the mean ± S.D. from three independent experiments. *P< 0.05, ****P< 0.0001

**Supplementary Figure. S2. To perform an autophagy flux with bafilomycin A1. a-b.** HCC cells were transfected with GV141-Vector and GV141-SOCS5, or transfected with siNC and siSOCS5 #3 or treated with DMSO (control) or bafilomycin A1 (100nM) or a combination of both treatments for 24 h. **a.** Western blotting analysis of LC3-II protein expression in Hep3B cells. β-actin was used as a loading control. b. Western blotting analysis of LC3-II protein expression in Huh7 and PLC/PRF/5 cells. β-actin was used as a loading control. The data are presented as the mean ± S.D. from three independent experiments. **P< 0.01, ***P< 0.001, ****P< 0.0001

**Supplementary Figure. S3.** Lentivirus infection rate in Huh7 cells from two groups.
