# Supplementary material for: SOCS5 inhibition induces autophagy to impair metastasis in hepatocellular carcinoma cells via the PI3K/Akt/mTOR pathway
Source: Cell Death Dis. 2019 Aug 13;10(8):612. doi: 10.1038/s41419-019-1856-y (PMC6690952; doi:10.1038/s41419-019-1856-y)
Supplement: Supplementary file 5 — Supplementary Table 1 [file 41419_2019_1856_MOESM5_ESM.doc]

**Supplementary Table 1.** Antibodies and primers

| **Antibodies** |  | |  | |  |  |
| --- | --- | --- | --- | --- | --- | --- |
| Name | Manufacturer | | Number | | Type | Usage |
| SOCS5 (31-L) | Santa Cruz | | sc-100858 | | Monoclonal | WB、IHC |
| SOCS5 | abcam | | ab97283 | | Polyclonal | IHC |
| PI3K p110β | Cell Signaling Technology | | #9655 | | Monoclonal | WB |
| p-AKT | Cell Signaling Technology | | #4060 | | Monoclonal | WB |
| AKT | Cell Signaling Technology | | #4691 | | Monoclonal | WB |
| p-mTOR | Cell Signaling Technology | | #5536 | | Monoclonal | WB |
| mTOR | Cell Signaling Technology | | #2983 | | Monoclonal | WB |
| p-ULK1 | Cell Signaling Technology | | #6888 | | Monoclonal | WB |
| ULK1 | Cell Signaling Technology | | # 8054 | | Monoclonal | WB |
| p-ATG13 | Rockland antibodies & assays | | 600-401-C49 | | Polyclonal | WB |
| ATG13 | Rockland antibodies & assays | | 600-401-C50 | | Polyclonal | WB |
| Beclin1 | Biowarld | | MB0030 | | Monoclonal | WB |
| P62 | Cell Signaling Technology | | #88588 | | Monoclonal | WB |
| LC3 | Cell Signaling Technology | | #12741 | | Monoclonal | WB |
| MMP9 | abcam | | ab38898 | | Polyclonal | WB、IHC |
| MMP2 | abcam | | ab37150 | | Polyclonal | WB、IHC |
| AFP | abcam | | ab46799 | | Polyclonal | IHC |
| β-actin | Sigma-Aldrich | | A3854 | | Monoclonal | WB |
| goat anti-mouse IgG-HRP | absin | | abs20001 | | Polyclonal | WB |
| goat anti-rabbit IgG-HRP | absin | | abs20002 | | Polyclonal | WB |
| DAPI | Beyotine Biotechnology | | C1002 | | **-** | IF |
| **siRNAs** | **sequences (5’-3’)** | |  | |  |  |
| SOCS5 #1 | CCCGAAUUGAGCAGUGGAATT/ UUCCACUGCUCAAUUCGGGTT | | | | | |
| SOCS5 #2 | CCACAGAAAUCCCUCAAAUTT/ AUUUGAGGGAUUUCUGUGGTT | | | | | |
| SOCS5 #3 | AACCAGUCAAGGCAAAGUATT/ UACUUUGCCUUGACUGGUUTT | | | | | |
| **shRNA** | **sequences (5’-3’)** | | | | | |
| shSOCS5 | AACCAGTCAAGGCAAAGTA/TACTTTGCCTTGACTGGTT | | | | | |
| **Primer sequences (5’-3’)** | |  | |  | |  |
| SOCS5 | F: ATTGATGGGCTCCCTCTACCC R: TGCCTTGACTGGTTCTCGTTCC PCR | | | | | |
| GAPDH | F: TGACTTCAACAGCGACACCCA R: CACCCTGTTGCTGTAGCCAAA | | | | | |

**WB: Western blotting; IHC: Immunohistochemistry; IF: Immunofluorescence; siRNA: Small interfering RNA**
